# Phase 2 Study of Intralesional PV-10 in Refractory Metastatic Melanoma

**DOI:** 10.1245/s10434-014-4169-5

**Published:** 2014-10-28

**Authors:** John F. Thompson, Sanjiv S. Agarwala, B. Mark Smithers, Merrick I. Ross, Charles R. Scoggins, Brendon J. Coventry, Susan J. Neuhaus, David R. Minor, Jamie M. Singer, Eric A. Wachter

**Affiliations:** 1 Melanoma Institute Australia and the University of Sydney, Sydney, NSW Australia; 2 St. Luke’s Hospital and Health Network and Temple University, Bethlehem, PA USA; 3 Queensland Melanoma Project, Princess Alexandra Hospital, University of Queensland, Woolloongabba, QLD Australia; 4 MD Anderson Cancer Center, Houston, TX USA; 5 University of Louisville, Louisville, KY USA; 6 University of Adelaide and Royal Adelaide Hospital, Adelaide, SA Australia; 7 California Pacific Medical Center, San Francisco, CA USA; 8 Provectus Biopharmaceuticals Inc, Knoxville, TN USA

## Abstract

**Purpose:**

This international, multicenter, single-arm trial assessed efficacy and safety of intralesional rose bengal (PV-10) in 80 patients with refractory cutaneous or subcutaneous metastatic melanoma.

**Methods:**

Sixty-two stage III and 18 stage IV melanoma patients with disease refractory to a median of six prior interventions received intralesional PV-10 into up to 20 cutaneous and subcutaneous lesions up to four times over a 16-week period and were followed for 52 weeks. Objectives were to determine best overall response rate in injected target lesions and uninjected bystander lesions, assess durability of response, and characterize adverse events.

**Results:**

For target lesions, the best overall response rate was 51 %, and the complete response rate was 26 %. Median time to response was 1.9 months, and median duration of response was 4.0 months, with 8 % of patients having no evidence of disease after 52 weeks. Response was dependent on untreated disease burden, with complete response achieved in 50 % of patients receiving PV-10 to all of their disease. Response of target lesions correlated with bystander lesion regression and the occurrence of locoregional blistering. Adverse events were predominantly mild to moderate and locoregional to the treatment site, with no treatment-associated grade 4 or 5 adverse events.

**Conclusions:**

Intralesional PV-10 yielded durable local control with high rates of complete response. Toxicity was confined predominantly to the injection site. Cutaneous bystander tumor regression is consistent with an immunologic response secondary to ablation. This intralesional approach for local disease control could be complementary to current and investigational treatments for melanoma.

Patients with regional metastatic melanoma (i.e., local, in-transit, and satellite recurrence) have a long median survival, but their clinical management can be challenging due to disease heterogeneity and frequent and persistent proliferation of lesions.^1^–^5^ Refractory lesions are often highly unpleasant for the patient; can lead to ulceration, bleeding, or infection; and can affect extensive areas for prolonged intervals before distant metastasis.^6^ Some patients with established visceral metastases also have dermal disease and similarly troublesome symptoms. Current treatment guidelines include surgical excision, local ablation, intralesional therapy, and regional chemotherapy, along with targeted drugs (e.g., against the V600E *BRAF* mutation).^7^ There are currently few drug therapies, especially for *BRAF* wild-type patients, that can provide rapid, sustained reduction of tumor burden with low toxicity.

Rose bengal disodium (RB) has been used as an intravenous liver function diagnostic and is still used as a topical ophthalmic diagnostic.^8^,^9^ PV-10 (a sterile, nonpyrogenic 10 % solution of RB in 0.9 % saline) is a small molecule agent for intralesional (IL) injection into tumors. After IL injection, PV-10 accumulates in tumor lysosomes resulting in rapid lysis of tumor cells.^10^ This primary ablative effect may induce a secondary tumor-specific T cell–mediated antitumor immune response.^11^,^12^ In a phase 1 study, a single IL dose of PV-10 was well tolerated, with a best overall response rate (BORR) at 12 weeks of 55 %. Notably, 3 of 11 patients had no evidence of recurrence for at least 28 months.^13^ Subsequently, this phase 2 study was undertaken, allowing repeat dosing of PV-10 and following patients for up to 52 weeks (clinical trial NCT00521053).

## Patients and Methods

### Study Design

Eighty patients were enrolled onto this international, multicenter, open-label, single-agent study. The governing institutional ethics committee for each study center approved the study protocol, and all patients provided written informed consent. Eligible patients had biopsy-proven confirmation of melanoma and at least one cutaneous or subcutaneous lesion ≥0.2 cm in diameter that could accurately be measured by ruler/caliper or ultrasound. They received a single IL injection of PV-10 to uniformly infiltrate each of up to 20 study lesions on day 0 (i.e.,≤10 target and ≤10 nontarget dermal lesions) using 0.5 mL PV-10 per cm^3^ of lesion volume. Treatment could be repeated at weeks 8, 12, and 16 for new nontarget lesions or existing target or nontarget lesions not exhibiting complete response. PV-10 was not injected into nodal or visceral lesions. One or two additional measureable, biopsy-confirmed dermal lesions could be designated for assessment of bystander response and were followed but not injected. Body mapping and digital photography with lesion identification markers and reference scale were used to accurately track all study lesions. Patients were followed for 52 weeks after initial PV-10 treatment (i.e., for at least 36 weeks after their last possible PV-10 treatment).

Study evaluations, including lesion photography, were performed at screening, on the day of PV-10 administration, at 1 day and 7 days after injection, and every 4 weeks thereafter during the treatment portion of the study (i.e., through week 16), and during long-term follow-up at weeks 24, 36, and 52. Radiologic assessments of visceral disease status were performed every 12 weeks throughout the study, and patients were transitioned into survival follow-up if at any time the investigator identified clinical or radiologic evidence of distant progression. No other melanoma therapy was permitted during the study interval. Treatment for concurrent or intercurrent illness, and wound care or management of pain, were allowed at each investigator’s discretion. Adverse events (AEs) were monitored over the study interval.

The study utilized a modified Fleming two-stage design.^14^ Interim assessment upon accrual of the first 40 patients required an objective response in at least eight patients to substantiate a true response rate between 10 and 30 %. Full accrual allowed testing of a projected 30 % primary efficacy rate (95 % confidence interval 20–40). Post hoc exploratory analyses were undertaken to understand results in certain subgroups.

### Criteria for Analyses

The primary end point of the study was BORR of target lesions, with secondary end points of progression-free survival (PFS) and duration of response, overall survival (OS), by-lesion BORR, and bystander lesion BORR, along with assessment of AEs, quality-of-life (assessed using the EORTC QLQ-C30 instrument), lesion pain (assessed via a 100 mm visual analog scale), and pharmacokinetics.^15^–^17^

**Fig. 1:**
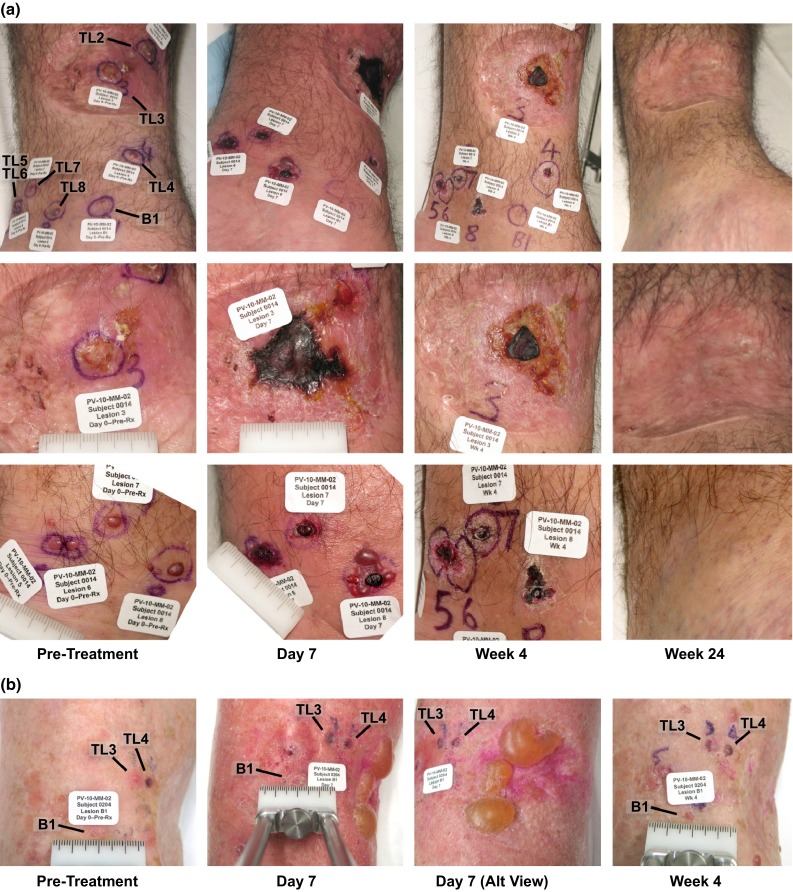
Example ablative effects of intralesional PV-10 in treatment-refractory melanoma. **a** Regional and close-up views of patient 0014, male, age 48, with stage IIIB melanoma of the lower extremity recurrent after 3 previous surgical interventions, treated once with 1.3 mL PV-10 into 10 target lesions on day 0 (bystander lesion B1 uninjected). All lesions were undetectable by ultrasound at week 24. Possible recurrence of target lesions TL2, TL3, TL4, TL8, TL9, and TL10 detected by ultrasound at week 36 (each 1–3 mm maximum diameter; none sampled) with TL9 and TL10 remaining at week 52 (2–3 mm) without further treatment. Extensive eschar of TL2 and TL3 within surgical scar is evident at day 7 with marked improvement by week 4. Small (grade 1) treatment-emergent perilesional blisters are evident in close-up view of TL8 at day 7. **b** Example of treatment-emergent blisters occurring in 40 % of the patient population and correlated with increased response rate of target lesions. Generally presenting as tense bullae 1–7 days after treatment, blisters typically resolved within 4 weeks with or without medication intervention. Close-up views of patient 0204, female, age 82 years, with stage IIIB melanoma of the lower extremity recurrent after four previous surgical interventions, treated once with 0.8 mL PV-10 into 8 target lesions on day 0 (bystander lesion B1 uninjected). Grade 2 blistering developed in a surgical scar medial to target lesions TL3 and TL4 within 4 days, with full resolution within 1 week without intervention

Response Evaluation Criteria in Solid Tumors (RECIST) was modified (mRECIST) to allow: (1) designation of cutaneous or subcutaneous lesions ≥0.2 cm in diameter as target lesions; (2) up to 10 cutaneous and subcutaneous target lesions to be followed; and (3) assessment of disease progression against baseline sum of longest diameters of target lesions, thereby allowing clinically insignificant progression (including locoregional appearance of new cutaneous or subcutaneous nontarget lesions) to be treated to week 16.^18^ Standard thresholds for percentage change in sum of longest diameters were used to define complete response (CR), partial response (PR), stable disease (SD), and progressive disease (PD). All lesions specified at baseline were followed over the course of the study with last observations carried forward for any lesions not measurable at a visit; response assessment was censored for missed visits. When eschar was reported, a standard query was used to ascertain lesion status. An example of measurable eschar that eclipsed baseline measurement is illustrated in Fig. 1a. To mitigate such artifacts and allow detection of regrowth after initial ablation, the first formal response assessment occurred at week 8 and was repeated at each visit thereafter. Patients failing to reach assessment at week 8 were deemed not evaluable and classified as PD.

## Results

### Patients

Eighty patients (intent-to-treat population, ITT; median age 70 years, range 33–97 years; Table 1) were enrolled over 19 months at 7 study centers: 3 in Australia and 4 in the United States. All patients had recurrent, locally advanced melanoma after a median of 6 previous interventions (range 1–19), and most had received multiple classes of treatment. The median time from initial melanoma diagnosis to initial PV-10 treatment was 42.3 months (range 1.7–752). Study lesions comprised a substantial total tumor burden with a median sum of longest diameters of 6.3 cm (range 0.9–21.0 cm), with a median of 7.5 study lesions per patient (range 1–22). The most prevalent comorbidity was hypertension (51 % of patients), followed by hypercholesterolemia (23 %) and depression (19 %).

**Table 1:** Patient characteristics

Characteristic	*N*	%
Gender		
Male	48	60
Female	32	40
Age		
<70 y	39	49
≥70 y	41	51
Disease stage		
III	62	78
IIIB	38	48
IIIC	24	30
IV	18	23
IV M1a	3	4
IV M1b	5	6
IV M1c	10	13
Treatment history		
Prior therapy		
Surgical excision	80	100
Nodal biopsy	50	63
Regional chemotherapy	19	24
Immunotherapy	17	21
Radiotherapy	17	21
Investigational agents	11	14
Systemic chemotherapy	10	13
Distal amputation	7	9
Other	6	8
No prior systemic therapy	45	56
Prior systemic therapy	35	44
Tumor burden in skin		
<10 lesions	44	55
≥10 lesions	29	36
Too numerous to count	7	9
ECOG status		
0	53	66
1	25	31
2	2	3

*ECOG* Eastern Cooperative Oncology Group

Patients received a mean of 1.8 PV-10 treatment cycles (median 2); 35 patients received a single cycle, and three patients received the maximum four cycles allowed by protocol.

### Efficacy

**Fig. 2:**
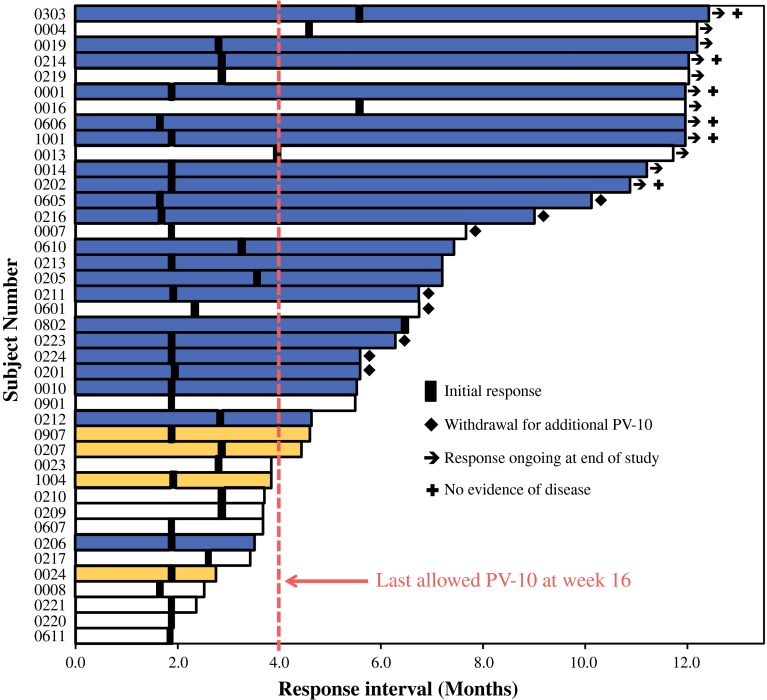
Temporal response of all responding patients (i.e., CR or PR in up to 10 cutaneous and subcutaneous target lesions). The 21 patients who experienced CR are shown in *blue*; the 16 stage III patients experiencing PR are shown in white; and the 4 stage IV patients experiencing PR are shown in *yellow*. Black bands indicate time of initial response. Patients who withdrew early to receive further PV-10 treatment under alternative protocols are designated with a *diamond*; patients with ongoing response at end of study interval are designated with an *arrow*; those with no evidence of disease at the end of the study interval are designated with a *plus* sign

Half of the ITT patients achieved an objective response in their target lesions (51 % BORR, 26 % CR + 25 % PR) (Table 2), with 8 % of patients having no evidence of disease after 52 weeks. Locoregional disease control (SD or better) was achieved in 69 % of patients, while 16 % were not evaluable as a result of progression before week 8. (This occurred predominantly in patients with extensive disease burden not injected with PV-10.) Onset of response occurred at a median of 1.9 months, corresponding with the first assessment of response. The median duration of response was 4.0 months (by RECIST) but was not met in the study interval when assessed by mRECIST. Time-to-event data for objective response are represented in Fig. 2, and PFS data for all patients are summarized in Table 2.

**Table 2:** Objective response of target lesions

			Patients categorized by disease burden at baseline							
Response of target lesion	ITT population		All lesions treated		Bystanders untreated		10 or fewer untreated skin lesions^a^		TNTC or stage IV	
No. of patients	80	%	28	%	26	%	7	%	19	%
CR	21	26	14	50	6	23	1	14	0	0
PR	20	25	6	21	8	31	1	14	5	26
SD	14	18	3	11	8	31	1	14	2	11
PD (PD + NEV)^b^	25	31	5	18	4	15	4	57	12	63
NEV: progression before week 8^b^	13	16	2	7	1	4	3	43	7	37
CR + PR	41	51	20	71^c^	14	54	2	29	5	26
CR + PR + SD (locoregional disease control)	55	69	23	82	22	85	3	43	7	37
Mean PFS, mo^d^	8.2		9.8^e^		8.9^f^		6.0		2.6	

*ITT* intent to treat, *TNTC* too numerous to count, *CR* complete response, *PR* partial response, *SD* stable disease, *PD* progressive disease, *NEV* not evaluable, *PFS* progression-free survival

^a^Median number of untreated lesions: 5

^b^Patients who were not evaluable were tracked separately but combined with PD for tabulation of outcome

^c^ *P* = 0.006 vs. TNTC or stage IV subgroup, BORR by *χ* ^2^ test

^d^PFS by mRECIST, maximum follow-up duration 12 months

^e^ *P* < 0.01 vs. TNTC or stage IV subgroup, by log-rank test

^f^ *P* = 0.04 vs. TNTC or stage IV subgroup, by log-rank test

Response rates by lesion among 491 target lesions in the ITT population were similar to those by patient, with 53 % of lesions achieving CR, 5 % PR, and 12 % SD.

Among the 42 patients with designated bystander lesions, 26 % experienced CR of their uninjected bystander lesions, 7 % PR, and 17 % SD. Response in bystander lesions strongly correlated with that of patients’ target lesions: CR or PR in target disease was associated with 56 % CR and 6 % PR in bystander lesions; in contrast, patients who did not experience an objective response of their target lesions had 6 % CR and 12 % PR of their bystander lesions (*P* = 0.023, BORR by *χ* ^2^ test).

Median OS was not reached for the ITT population. Mean OS for stage III patients was >12 months (89 % 1-year survival, median not reached), while for stage IV patients, median survival was 6.5 months (39 % 1-year survival).

Because the protocol limited PV-10 treatment to the first 16 weeks, 11 patients subsequently withdrew to receive further PV-10 treatment under alternative protocols.

### Safety

All patients experienced one or more AE during the study (Table 3); most were grade 1 or grade 2, while 15 % of patients had at least one grade 3 AE deemed at least possibly related to study treatment. The most common AEs occurred at the injection site, led by transient pain (reported by 80 % of patients), edema (41 %), and vesicles (39 %). Six patients experienced mild (4 %) or moderate (4 %) injection site photosensitivity, and one (1 %) experienced a severe generalized photosensitivity reaction. All of these resolved without sequelae. No life-threatening or fatal AEs at least possibly related to the study treatment were reported.

**Table 3:** Most frequent adverse events at least possibly related to study treatment

System organ class and preferred term^a^	Adverse events^b^ (ITT population, *N* = 80)				
	CTCAE grade			Total	%
	1	2	3		
General disorders and administration site conditions					
Injection site pain	29	25	10	64	80
Injection site edema	19	14	0	33	41
Injection site vesicles	17	13	1	31	39
Injection site discoloration^c^	13	12	0	25	31
Injection site swelling	14	7	1	22	28
Injection site pruritus	14	3	0	17	21
Injection site erythema	6	4	1	11	14
Injection site infection	3	2	1	6	8
Injection site inflammation	0	6	0	6	8
Injection site photosensitivity reaction^d^	3	3	0	6	8
Injection site ulcer	4	1	0	5	6
Peripheral edema	3	0	1	4	5
Fatigue	3	1	0	4	5
Injection site rash	4	0	0	4	5
Injection site warmth	2	2	0	4	5
Lethargy	3	1	0	4	5
Injection site cellulitis	0	2	1	3	4
Gastrointestinal disorders					
Diarrhea	5	0	0	5	6
Nausea	2	3	0	5	6
Dysphagia	0	0	1	1	1
Nervous system disorders					
Headache	11	2	0	13	16
Skin and subcutaneous tissue disorders					
Photosensitivity reaction^d^	0	0	1	1	1

*ITT* intent to treat, *CTCAE* Common Terminology Criteria for Adverse Events

^a^System organ class and preferred term are based on the MedDRA version 13.0 terminology dictionary. Locoregional adverse events were coded to “injection site” preferred terms to differentiate these from systemic events

^b^Includes all AEs with an incidence of 5 % or higher and all CTCAE grade 3 and higher AEs; there were no treatment-related grade 4 or 5 AEs reported. If a patient experienced an AE more than once during the study, the greatest severity is presented

^c^Discoloration locoregional to injected lesions

^d^Combined incidence of injection site and skin and subcutaneous tissue photosensitivity reactions: 9 %

Quality-of-life assessment throughout the study interval showed no substantial change from baseline on any quality-of-life dimension after PV-10 treatment. Pain scores indicated transient pain in treated lesions during the first week after treatment that generally resolved to baseline within 4 weeks. Approximately 60 % of patients received local anesthesia at the time of PV-10 injection.

### Pharmacokinetics

Pharmacokinetic data were consistent with the literature on RB and illustrate PV-10 clearance via an apparent biexponential process, with a rapid initial distribution/absorption phase occurring during the first 24 h (k_D/A_ = 0.0020 min^−1^, t_1/2,D/A_ = 5.9 h), followed by slower terminal elimination (k_E_ = 0.00012 min^−1^, t_1/2,E_ = 100 h).^19^ Relatively low uptake during the initial phase (geometric mean  %AUC∞ = 17.3 %) and intercepts for the initial and terminal phases (427 and 73 ng/mL, respectively) are consistent with prolonged retention of drug in injected lesions, with potentially significant systemic exposure only during the initial phase.

### Untreated Disease Burden

Although only cutaneous and subcutaneous lesions were injected, the study enrolled patients with stable visceral disease (including brain, lung, and liver metastases) who also had injectable dermal disease. To test for a relationship of response to untreated disease burden, patients were classified according to their reported tumor burden at baseline: all known disease treated with PV-10 (28 patients); all known disease treated with PV-10 (with the exception of 1–2 uninjected bystander lesions) (26 patients); 10 or fewer untreated and unmeasured dermal lesions (7 patients); and dermal disease burden classified as too numerous to count or as stage IV disease (19 patients). For the 54 patients in the first two subgroups, their study lesions, including bystander lesions, represented all documented disease at baseline. These patients, who received PV-10 to all or substantially all of their disease burden, achieved markedly higher response rates compared to patients with substantial untreated disease burden (Table 2).

### Locoregional Blistering

Another exploratory analysis was undertaken to understand the relevance of locoregional vesicles (blistering). Transient, fluid-filled bullae were observed in 40 % of patients (23 % grade 1, 16 % grade 2, and 1 % grade 3, including one occurrence reported as possible grade 1 photosensitivity), both perilesional and locoregional and treatment emergent within 1–7 days of PV-10 administration. Onset was independent of dose with no apparent relationship to prior PV-10 administration (Fig. 1). These generally resolved within 4 weeks, with or without medication intervention and without long-term sequelae; blistering was associated with a marked increase in response: 44 % of patients with blisters experienced CR, versus 15 % without blisters (*P* = 0.008).

### Age, Sex, Treatment History, and Investigator

Exploratory analyses were undertaken to assess relevance of demographics, treatment history, and investigator. Equivalent target lesion responses occurred in patients above and below the median age (54 vs. 49 % BORR, *P* = 0.8) and for men and women (58 vs. 41 %, *P* = 0.2). Dichotomization according to treatment history (systemic or regional chemotherapy or immunotherapy vs. naive patients) showed no significant difference in target lesion response (43 vs. 58 %, *P* = 0.3). Similar response rates were observed across study centers, with all centers reporting at least one patient experiencing PR or better (Fig. 2), and with five of the seven centers reporting patients with no evidence of disease after 12 months. Patterns of AEs were also similar across centers.

### Visceral Disease

Although the study was not designed to quantitatively follow lesions in visceral organs, comprehensive radiologic imaging provided some insight into whether PV-10 could have an impact on visceral disease. While a substantial fraction patients with stage IV disease were classified as not evaluable due to early progression (Table 2), four patients experienced SD or PR of their visceral disease (including patients with multiple pleural and hepatic metastases); three of these patients also exhibited SD or better outcome in their target lesions, similar to the correlation of bystander response to that of target lesions. Seven stage IV patients survived through the end of the 52-week follow-up interval, including 4 of 10 patients with M1c disease.

## Discussion

Patients enrolled onto this study had treatment-refractory cutaneous and subcutaneous metastatic melanoma accessible to injection and were not candidates for systemic therapy as a result of age, comorbidity, refractory disease, or drug unavailability; one quarter had visceral metastases plus injectable disease of the skin.

The primary ablative effect of PV-10 is evident upon minimal intervention (Fig. 1a) and illustrates potentially rapid durable disease control (Fig. 2). The predominantly locoregional AE profile contrasts with global morbidities reported for many systemic and emerging local therapies.^20^–^28^ Response typically occurred after one or two PV-10 treatment cycles versus six or more cycles for other recent investigational IL therapies, with response observed in treated and untreated disease.^29^–^32^

Untreated tumor burden had a major impact on response: patients receiving injections to most or all tumor deposits exhibited high rates of durable response, a trend that may signify abrogation of immunosubversion by untreated tumor burden.^33^–^35^ Simultaneous reduction of tumor burden and immune stimulation with PV-10 has proven powerful in animal models of metastasis, and correlation of response in injected and uninjected disease in this and previous clinical studies is consistent with such results.^11^,^13^ Emerging evidence of a functional immune response secondary to ablation (including increased levels of cytotoxic CD3^+^ and CD8^+^ T cells in peripheral blood of patients refractory to immune checkpoint inhibitors and targeted therapies) bolsters the potential relevance of the observed bystander effect.^12^ Association of improved outcome with locoregional blistering suggests that this AE deserves further investigation.

Three patients from this study experienced unexpectedly positive responses upon subsequent radiotherapy of previously injected, uninjected, and new lesions.^36^ This suggests that the combination of PV-10 with other treatments may have merit in advanced-stage disease with substantial tumor burden inaccessible to injection. In particular, the tumor-specific immune stimulation resulting from PV-10 ablation is potentially additive or synergistic with nonspecific immunotherapies.^12^,^37^

In summary, intralesional PV-10 elicited robust and durable tumor regression in refractory cutaneous and subcutaneous melanoma with transient locoregional toxicity. The primary ablative effect of PV-10 reduced the size of injected tumors quickly, while regression of uninjected bystander lesions is consistent with a secondary immune response. These data suggest that PV-10 has utility in the management of melanoma patients with injectable cutaneous and subcutaneous disease. Future studies will comprehensively assess the effect of PV-10 on PFS to document potential longer-term benefits of locoregional disease control.
